# A bibliometric analysis of the 100 most-influential papers in the field of anti-diabetic drugs

**DOI:** 10.2144/fsoa-2023-0230

**Published:** 2024-05-15

**Authors:** Fatima Azam, Muhammad Hamza Dawood, Aroosa Roshan, Maryam Urooj, Zoha Khan, Muhammad Omar Larik, Fatima Mustafa Lakdawala, Ahad Yousuf Moulvi, Ifrah Salim, Mehak Abbas Zaidi, Alizeh Imran

**Affiliations:** 1 Dow International Medical College, Dow University of Health Sciences, Karachi, Sindh, 74200, Pakistan; 2 United Medical & Dental College, Karachi, Sindh, 75190, Pakistan; 3 Ziauddin Medical College, Karachi, Sindh, 75000, Pakistan

**Keywords:** antidiabetic drugs, bibliometric analysis, biomedical trends, diabetes, gender bias

## Abstract

**Aim:** We analyzed the 100 most-cited articles on all anti-diabetic drugs. A comprehensive literature review found no bibliometrics on this. **Methods:** Two researchers independently extracted articles from Scopus and ranked them by citation count as the ‘top 100 most-cited’. **Results:** The median number of citations is 1385.5. Most articles are from the USA (n = 59). Insulin has the most papers (n = 24). Majority (n = 76) were privately funded and contained at least one conflict of interest (n = 66). The New England Journal of Medicine has the most publications (n = 44). Male authors made majority of both first and last authorship positions. **Conclusion:** This study aims to aid in directing future research and in reducing biases.

## Abstract (plain language summary)

The 100 most cited anti-diabetic original articles were published between 1971–2022 from a total of 46 nations.

The highest frequency of articles occurred between 2006–2010 (n = 27).

The median number of citations was 1385.5, ranging from 774 to 22,496.

Authors from 46 nations contributed to this list; however, more than half of these articles were from the USA (n = 59), followed by the UK (n = 31) and Canada (n = 24).

Insulin had the most papers published (n = 24).

*The New England Journal of Medicine* (n = 44) and *The Lancet* (n = 18) have contributed most to the publications.

The analysis also highlighted a gender disparity in first and senior authorship positions, with male authors predominating in anti-diabetic research.

Most articles (n = 76) were funded privately, followed by publicly funded (n = 49).

The average number of authors with a conflict of interest was 5.23, and 66/100 publications contained at least one.

Diabetes is a widespread problem in both the developed and developing world, with numerous therapeutic contexts calling for anti-diabetic medications, including lowering blood sugar levels, managing weight loss, and reducing mortality from heart disease [^1-3^]. As a result, anti-diabetic medications have become an essential toolkit for various clinicians to treat this widespread disease and its associated conditions in every part of the world. A few bibliometrics on diabetes and metformin, a diabetic treatment, have been published [^4-7^]. None, however, shed light on all anti-diabetic medications.

Bibliometric analysis integrates collection and metrics to analyze dynamic literature information and citation trends. Citation metrics measure an article's impact over time based on how often it's cited. Academic achievement relies heavily on producing highly cited papers [8]. The results from this simple analytical tool can aid researchers in learning about trends and the scope of research orientation within a particular domain. This effectively guides the emergence of creative topics [9], in addition to identifying and working with experts and impactful collaborators. This tool also allows governing bodies to direct funds toward areas that require research attention [10]. It is widely used to analyze trends, impact, research productivity and interventions in fields such as neurology [11], psychiatry [12] and oncology [13].

Biomedical research has grown exponentially, complementing the surge in evidence-based medicine [^14-16^]. This has enormously elevated scientific understanding. Researchers, institutions, and even countries around the globe compete to contribute to valuable medical research. Historically, scientific research evaluation is a challenging endeavor. The necessity to acquire pertinent knowledge and rivalry for empiricism ignite a type of ranking that distinguishes the most influential publications within a discipline, among authors and their countries of origin. Bibliometric analysis accomplishes this aim. This study aimed to identify and analyze the 100 most cited and influential original anti-diabetic medication articles across all specialties.

## Materials & methods

### Bibliometric approach

In October 2022, we performed a literature search to identify the 100 most cited articles on anti-diabetic drugs to conduct a bibliometric analysis. Elsevier's Scopus database was used to retrieve the relevant articles for the analysis, as it indexes a wider spectrum of journals compared with other databases like PubMed, Web of Science and Google Scholar. No particular time interval or limitation was set for the search and retraction. To ensure an extensive review of the literature available on the topic, studies on non-human subjects, studies without abstract availability and studies in languages other than English were all part of the inclusion criteria during the database search.

### Search strategy

Our first step was to build a valid search strategy that would help retrieve as many relevant documents as possible, and the main search terms included “hypoglycemic”, “anti-diabetic” and “SGLT2”. The keyword search was also expanded to include the names of individual anti-diabetic drugs as keywords. These names were derived primarily from a recent study on oral hypoglycemic medicines. Major keywords used included “repaglinide” OR “nateglinide” OR “glipozide” OR “glyburide” OR “glimepiride” OR “rosiglitazone” OR “pioglitazone” OR “sitagliptin” OR “acarbose” OR “miglitol” [17]. Furthermore, the SGTL2 inhibitors were obtained from the classification given in Usman *et al.* systematic review and meta-analysis study [18]. In addition, we used PUBMED's mesh terms to ensure a comprehensive search string was made that included the pharmaceutical, generic and trade names of all the anti-diabetic drugs. The full search strategy can be available upon request and has not been mentioned in the context due to space limitations.

### Top 100 articles on anti-diabetic drugs

Two independent reviewers (F.A. and A.R.) individually searched Scopus, blinded by each other's findings, to extract relevant original articles from the database. No review, meta-analysis or guideline was included in the top 100 list. The retrieved studies were primarily focused on anti-diabetic drugs. The abstracts for all articles retrieved during the research were then reviewed to determine their relevance and inclusion. Each reviewer retrieved a separate list of the 100 most cited articles, which was later compared for the disparity, and articles that both reviewers selected after consultation and agreement on their relevance and inclusion were added to the final list. This was done to reduce biases in the findings. Any confusion or uncertainty was addressed through discussion between the two reviewers, and a third party (M.U.) was consulted when necessary. Then, using Scopus, the articles in the final list were organized using the ‘cited by’ option and were compiled in a descending order.

### Statistical analysis

The list of the retrieved top 100 articles was then analyzed using manual screening via Scopus, and were evaluated for specific characteristics, including: 1) title, 2) citations 3) citations per year 4) year of publication, 5) journals that published >1 article and their metrics i.e., impact factor (IF), Eigenfactor score (ES), CiteScore, Source Normalized Impact per Paper (SNIP), SCImago Journal Rank (SJR) and Quartile (from Q1 to Q4), 6) country of origin, and 7) names of authors and their affiliations. The studies were categorized into different study types too by reviewing the methodology section of each article. In cases where clarification was required, the results and introduction sections were also examined to confirm the study type. Any confusion or uncertainty was addressed through discussion between the two reviewers, and a third party (M.U.) was consulted when necessary. For the compilation of the list, we considered all types of original articles. By quantifying these different types, we aimed to identify which kinds of original articles had a greater representation in the top 100 list. The metrics of the journals (IF, ES, CiteScore, SNIP, SJR and Quartile) that produced articles in the top 100 list previously identified are used to determine which journals are more prestigious, with journals that have a higher score on these metrics are generally regarded as more reputable than those with lower scores [19]. The presence of conflict of interest (COI) was assessed within the articles. In cases where there was no dedicated COI section, a disclosure section was located either at the bottom of the article or alongside it within a separate disclosure and interests document, where authors explicitly disclosed any conflicts of interest. This included providing details about the nature, source, and occurrence of the COI. Values like mean, median, and interquartile range (IQR) were calculated for the total number of citations and citations per year, and Pearson's correlation coefficient was also calculated with the 95% confidence interval to check for any correlation between a journal's IF and the number of articles in the list. A p-value of <0.05 was considered significant for the statistical test. A second search using the same search string and analysis techniques was also conducted in order to retrieve the list of the 10 most-cited review, systematic review, and meta-analysis articles and the ten most-cited guidelines on anti-diabetic drugs. All tables and charts were made using Microsoft Word and Excel, and SPSS version 23.0 (IBM Corp., Armonk, NY, USA) was used to conduct all statistical and significance tests.

### Data availability

The data associated with the paper was collected from the database named Scopus, which are summarized below in the form of tables and figures.

## Results

### Citations counts, citations per year & citation trends

**Figure 1.**
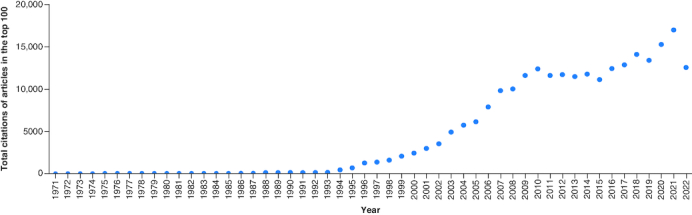
Total citations of articles in the list each year.

Supplementary Table 1 lists the top 100 articles with their citation counts and citations per year in the field of anti-diabetic drugs. The total number of citations for 100 articles summed up to be 241,054. The number of citations for these articles ranged from 774 to 22,496 with a mean of 2410.54, and a median of 1385.5 (IQR = 1249.25). Approximately 3.5% of the total citations were self-citations. The number of citations per year ranged from 59.5 to 775.7, with a median of 96.45 and IQR of 167.875. Figure 1 shows the trend of total citations for the 100 articles in the list by year. There was a rapid rise in the total citations beginning from the year 1994, first peaking in the year 2010. Minor fluctuations were then observed, with a small dip in 2015, after which a rapid rise was again seen following the years 2016–2021 and eventually a sharp decline in the number of total citations in the year 2022.

### Year of publication, origins & authorships

**Figure 2.**
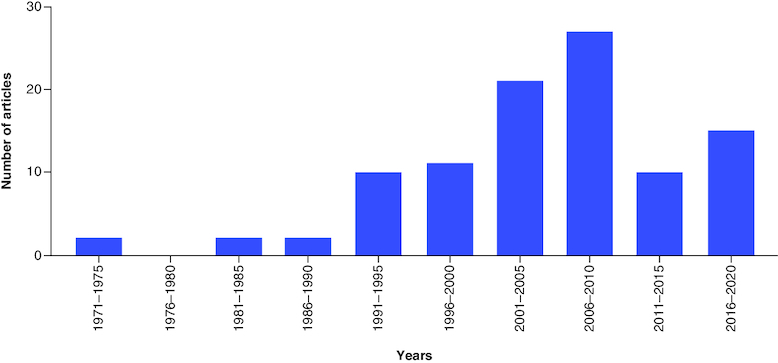
Number of publications in each 5-year interval.

**Figure 3.**
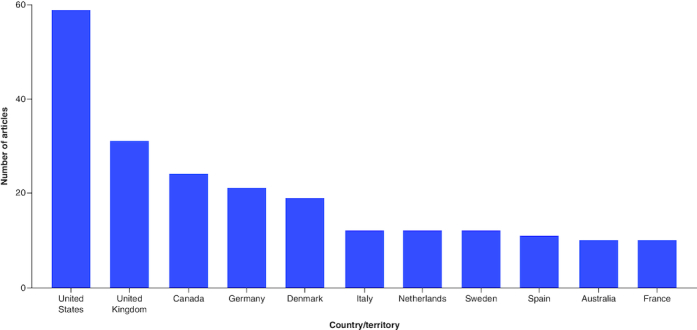
Countries with ten or more articles in the top 100 list.

The 100 most cited articles were published between 1971 and 2022. During this 51-year span, the largest number of these articles (n = 15) were published in the 5-year period from 2016 to 2022, as highlighted in Figure 2. The 100 most cited papers originated from 46 countries, with 56% of the articles having authors from more than one country. The three most popular countries of origin were the USA (n = 59), followed by the UK (n = 31), and then Canada (n = 24), as highlighted in Figure 3. A total of 10,445 authors contributed to these 100 articles, with each having a median of 8.50 authors (IQR = 12). Authors with ≥5 articles in our 100 most cited articles have been listed in Table 1. The greatest number of most cited articles were coauthored by Rosenstock, J, numbering 9 articles, followed by Zinman, B, (n = 9), Woo, V (n = 8) and Lachin, JM (n = 7).

**Table 1.** Authors with ≥5 articles in the top 100 list.

Study	Publications (n)
Rosenstock, J	9
Zinman, B	9
Woo, V	8
Lachin, JM	7
Buse, JB	6
Gerstein, HC	6
Hanefeld, M	6
Holman, RR	6
Leiter, LA	6
Riddle, MC	6
Burgess, L	5
Cartasegna, L	5
Conway, J	5
Dumas, R	5
Holst, JJ	5
Hramiak, I	5
Lochnan, H	5
Petit, C	5

### Journal & institutional affiliations

The 100 most cited articles were published among 24 different journals, with the top four producing around three-fourths of the articles. These top four journals included (1) *The New England Journal of Medicine*, (2) *The Lancet*, (3) *Diabetes Care* and (4) *Diabetes*. All journals that had more than one article in the top 100 were in Q1 (Quartile range is from Q1–Q4). The IFs of the top four journals ranged from 9.337 to 176.079. We found a statistically significant association between the journal IF and the number of articles in the most cited 100 list (p = 0.024). Journals with >1 article in the most cited 100 articles have been listed in Table 2. A total of 160 institutions were affiliated with the articles in our list. The University of Toronto led with 16 publications, and Harvard Medical School and Saint Michael's Hospital University of Toronto followed closely behind. Table 3 lists the institutions having ≥6 articles on our list.

**Table 2.** Journals with >1 article in the list.

Name of journal	Articles (n)	Impact factor	Eigenfactor	CiteScore	SNIP	SJR
*New England Journal of Medicine*	44	176.079	0.682	134.4	17.194	26.015
*Lancet*	18	202.731	0.407	133.2	25.787	14.607
*Diabetes Care*	7	17.152	0.117	27.8	4.781	6.008
*Diabetes*	5	9.337	0.087	12.3	1.654	2.635
*Diabetologia*	3	10.46	0.053	17.6	2.486	3.349
*British Medical Journal*	2	93.333	0.162	15.3	8.667	2.867
*Circulation*	2	39.918	0.269	42.1	6.144	7.800
*Journal Of Clinical Investigation*	2	19.456	0.185	23.7	2.423	5.117
*Nature*	2	69.504	1.443	83.4	11.591	20.957

**Table 3.** Institution affiliated ≥6 articles in the list.

Name of institute	Articles (n)
University of Toronto	16
Harvard Medical School	10
Saint Michael's Hospital University of Toronto	10
University of Oxford Medical Sciences Division	8
Novo Nordisk A/S	8
The George Washington University	7
Oregon Health & Science University	7
UNC School of Medicine	7
Københavns Universitet	7
University of Glasgow	6
Mount Sinai Hospital of University of Toronto	6
Imperial College London	6
Brigham and Women's Hospital	6
University of Texas Health Science Center at San Antonio	6
UT Southwestern Medical Center	6

### Top drug classes for anti-diabetic drugs

The top anti-diabetic drug classes have been listed in Table 4. Insulin takes the lead on the list (n = 24), closely followed by articles with >1 drug class (n = 23), and then incretin mimics (GLP-1 Agonists/analog) (n = 17), and the rest follow. Supplementary Figures 1–3 show the number of publications in 5-year intervals for the previously mentioned most-cited drug classes, respectively. Papers published on insulin peaked twice, in 1991–1995 and in 2001–2005 as shown in Supplementary Figure 1, but has been in steady decline post-2005 with no top articles published after 2015. Papers published on >1 drug class had the highest peak at nine top papers published in 2006–2010 as seen in Supplementary Figure 2. As seen in Supplementary Figure 3 there has been a rise in top papers published on incretin mimetics (GLP-1 agonists/analog) with the most papers published recently in 2016–2020.

**Table 4.** Top anti-diabetic drug classes.

Drug class	Number of articles
Insulin	24
>1 drug class	23
Incretin mimetics (GLP-1 agonists/analog)	17
SGLT2i	9
Thiazolidinediones (TZDs)	9
Biguanides (including metformin)	8
DPP4i	4
Sulphonylurea	3
Alpha-glucosidase inhibitors (AGIs)	2
IL-1 receptor antagonist	1

### Sponsors that funded ≥6 articles

Table 5 lists the total number of institutes that funded ≥6 articles, with the National Institute of Diabetes and Digestive and Kidney Diseases being the largest funding sponsor, followed by the National Institutes of Health, and Novo Nordisk.

**Table 5.** Institutes that funded ≥6 articles.

Funding	Number of articles
National Institute of Diabetes and Digestive and Kidney Diseases	15
National Institutes of Health	7
Novo Nordisk	7
AstraZeneca	6
National Center for Advancing Translational Sciences	6
National Center for Research Resources	6
National Eye Institute	6

### Classification of articles based on study type

Among the 100 articles, most were randomized control trials (RCTs) (n = 84), and the least were animal RCTs (n = 1). Moreover, two cross-sectional, four experimental trials, six animal experimental trials and three prospective studies were conducted, as shown in Supplementary Table 2. All of the top ten articles of the top 100 most-cited list were RCTs.

### Funding

Almost all articles received funding (n = 96), and Table 6 shows that the majority of these articles were funded privately (n = 76), followed closely by publicly funded articles (n = 49). When looking at the trends within the top ten of the top 100, nine articles are privately funded and eight are publicly funded. One out of the top ten articles is only publicly funded. Out of the top ten, only three are funded by non-profit organizations.

**Table 6.** Types of funding received by & number of conflict of interests (COI) reported by articles.

Funding (out of top 100)	Articles (n)
Total	
Private	76
Public	49
Non-profit	31
None	4
Only funded by one type	
Only privately funded	37
Only publicly funded	9
Only non-profit funded	4

Conflict of interest (COI)	Articles (n)
Number of articles with at least one COI	66/100
Mean number of authors with COI in an article	5.23
Median number of authors with COI in an article	2
Minimum COI of an article	0
Maximum COI of an article	39

Moreover, some of the articles in the list were only funded by one type of funding as seen in Table 6, however, most articles had a variety of funding (i.e. privately and publicly, publicly and non-profit, all three), therefore the values presented in Table 6 do not add up to 100 since many of the articles overlapped in the type of funding they received.

### Conflict of interest (COI)

Two-thirds of the articles had at least one COI (n = 66), as shown in Table 6, with a mean number of authors (n = 5.23), and a median number of authors (n = 2). Out of the top 100 articles, the top 2 have no COI. Three articles in the top ten have one COI reported and four have five or more COI reported.

### Gender distribution

**Figure 4.**
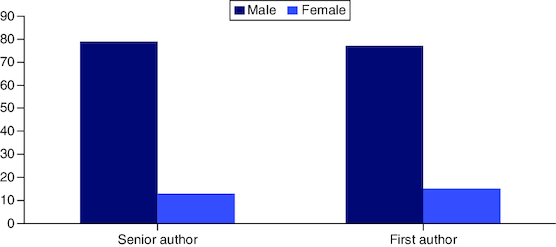
Gender distribution based on first and senior authorship within the top 100 list.

Among the 100 articles, there is a significant gap between the percentage of women and men as first authors and senior authors in our study. The majority of both senior and first authors were male as depicted in Figure 4. As shown in Supplementary Table 3, 86% of senior authors were male; similarly, 84% of the first authors were also male. Out of the top 100, 28 of the first and senior authors were female. Eight out of the 100 articles were not included in the gender analysis, because the studies were conducted by large groups hence, lacked first and last authors. Out of the top ten articles, five were reported to have both the first author and the senior author as male. Within the top ten, there were two female first authors and one female senior author.

### Review articles & cited guidelines

Supplementary Table 4 shows the ten most-cited review, systematic review and meta-analysis papers in the field of ‘anti-diabetic drugs’ along with their total citations, and the average number of citations per year. Supplementary Table 5 shows the ten most cited guidelines in the field of ‘anti-diabetic drugs’, along with their total citations and the average number of citations per year.

## Discussion

This analysis has revealed the innovative research being conducted in the field of antidiabetic drugs. This trend can be justified by the increasing prevalence of diabetes mellitus (DM) globally, with global prevalence nearly doubling within the last 30 years [20]. Moreover, this rise is expected to continue, with almost 10% of the global population projected to be victims of DM by the year 2040 [21]. Although the peak may have passed, the increasing demand for treatment in both high- and low-income countries [20] will continue to propel research in the field of anti-diabetic therapies, and it is anticipated that this industry will retain its relevance in the years to come. The rise in cases of DM can be explained by the concurrent rise in obesity worldwide. Numerous studies have illustrated a strong correlation between increasing ‘body mass index’ with an increasing prevalence of Type II diabetes mellitus (T2DM) [22,23].

The top journals publishing the most-cited research on anti-diabetic drugs include ‘*The New England Journal of Medicine*’ and ‘*The Lancet*’. It can be observed that some authors had exhibited a preference toward journals specialized in the study of DM and/or endocrinology; however, the majority of the top-cited articles had been published in generalized high-impact factor journals receiving a broad readership. Additionally, a weak relationship was observed between the journal's IF and the most-cited articles published within the journal. This challenges the concept of articles published in high-impact factor journals receiving more citations than those published in low-impact factor journals, particularly with research conducted on anti-diabetic drugs.

It is interesting to note that although metformin is considered the first-line therapy for DM, a large number of articles have highlighted incretin mimetics (GLP-1 agonists/analog) after insulin. This could be due to the newly discovered role of incretins in the treatment of pre-diabetes, which have been shown to delay B-cell failure and aid in weight loss [24]. Another explanation could be the increasing number of trials highlighting the cardiovascular benefits of GLP-1 agonists in patients with T2DM [25]. Expectedly, many articles have mentioned more than one class of drug for the treatment of DM, as the majority of anti-diabetic therapies are dual therapies [26]. Scarce mention of acarbose and sulfonylureas may suggest decreased clinical use, warranting further investigation into the efficacy of such drugs, and the cause of their faltering role in treating DM. The highly undesirable safety profile and extensive contraindications of acarbose and sulfonylureas, especially when compared with incretin mimetics, may be responsible for the decreased research interest observed within the field of anti-diabetic therapies [27,28].

As highlighted previously, the top 10 countries associated with the research of anti-diabetic therapies are highly developed, wealthy nations. This is a deeply concerning trend, as the disease is no longer confined to the wealthy, with numbers in less developed communities increasing faster than ever before [21]. Diabetes has also been shown to target specific ethnic groups, whom may benefit from targeted therapies developed due to increased inclusivity in research [21]. There were signs of this trend reversing with certain nations, such as India, becoming active players within the field, which would result in the market for anti-diabetic therapies becoming more inclusive, holistic and effective as the world battles against the burden of this global disease [20].

A concerning trend was the lack of authorship positions obtained by female researchers. Numerous reasons may explain this trend, ranging from the lack of mentorship and institutional opportunities offered to the differences in the roles and responsibilities of women in their family lives [29]. Moreover, males are also more likely to receive funding for anti-diabetic research, further challenging the contribution of female researchers in this industry [30]. Additionally, female researchers have voiced their feelings on the lack of belonging and inclusion, accompanied by a heightened perception of bias specifically directed toward them in academic settings [31]. The disparity between the genders of diabetes researchers could hinder advancement toward gender-specific and personalized treatment for diabetes, as an increase in female researchers would inevitably increase the inclusion of women in clinical trials, resulting in an enhanced understanding of the pathophysiology of the disease [28]. The gender gap may be improved by implementing various strategies to overcome the barriers to research. This can be done by providing funding through gender-neutral award programs, in which a portion of funds can be used for childcare, or hiring additional personnel to assist in the research process. The unconscious gender bias that exists in institutions may also be reduced by issuing statements and taking action by addressing ‘implicit bias’, which would reiterate the need for gender equality [32]. Furthermore, pre-invited female speakers in academic conferences should be aligned with the overall percentage of women in the field of endocrinology, to increase their recognition, while also improving the dissemination of their research [33]. Recent reports, however, say that most of the next generation of endocrinologists will be women, so it is possible that these trends will change in the near future [29].

The largest funding organizations for the top-cited articles are ‘National Institute of Diabetes and Digestive and Kidney Diseases’, with the ‘National Institute of Health’ and ‘Novo Nordisk’ being the second largest sponsors. With up to seven different sponsors involved in funding six or more most-cited articles, this provides a valuable insight that anti-diabetic drugs will receive greater sponsorship and funding in the future, with the global T2DM market size expected to double to $62 billion by 2030 [34]. Although it is important to consider the possibility of bias that may exist between industry sponsored research and non-industry sponsored research, where industry sponsored studies may be expected to reveal more promising and favorable results. However, it was observed that there was no significant increase in the reported favorable outcomes when industry-sponsored, and non-industry-sponsored research were compared. This held true for anti-diabetic drugs including metformin and glipizide; however, a significant increase was observed in the favorable outcomes reported in research involving pioglitazone [35].

Our bibliometric analysis also featured the various conflicts of interests (COI) of each of the top 100 most-influential articles. In our bibliometric analysis, the methodological approach employed in the detection of any potential COI included any executive positions, extra-institutional affiliations, or stocks/financial conflicts relevant to any organization or company. An example includes “*Dr. Hamman owns stock in Bristol-Myers Squibb, which sells metformin in the United States*”, in a study conducted by Knowler *et al.* [36]. Another example includes “*Dr. Bouillon holds a J. J. Servier Diabetes Research Chair*”, in a study conducted by Van der Berghe *et al.* [37]. The purpose of including such information within this analysis aids us in highlighting potential bias within the list of most-influential articles. There are numerous implications arising from the presence of COI in published research. It is thought to obstruct the formation of effective policies leading to healthcare improvement, and may be pervasive in influencing medicine regulation, implying a generalized bias and influence within the field of medicine [38]. Moreover, multiple reports have indicated the presence of selective reporting and a subsequent increased favorability of clinical outcomes, with greater efficacy and fewer risks observed in industry-sponsored research in comparison to non-industry sponsored research [39]. In summary, the presence of COI has been shown to influence the study design, the study outcomes and the relevant statistical analyses employed [40]. There are various management strategies in minimizing the negative effects of COI, including: (i) disclosure of conflicts to an individual's institution, peer-reviewers, and publications, (ii) prohibition from research participation in areas of strong conflicts, and (iii) intensified scrutiny of study design from institutional review boards (IRB), research supervisors and public research registration bodies [40,41].

The thorough and diligent efforts of all clinicians who conducted original research and clinical trials have been summarized via review articles, which have been listed in Supplementary Tables 1 & 4. These publications will aid current and future generations of physicians, as they venture to establish international guidelines for the treatment of diabetes.

## Implications & future directions

This bibliometric analysis revealed the dynamic nature of the research being conducted in the field of anti-diabetic drugs. With the rising prevalence of diabetes mellitus worldwide, it becomes essential to explore the ongoing efforts conducted by various physicians and researchers across the world [22]. Therefore, it was reasonable to discover that the greatest number of highly-cited and most-influential articles had been published within the period of 2016–2022, exemplifying the global efforts being made toward treating and managing diabetes mellitus. However, the male-dominated field of anti-diabetic research is a concerning trend, and may lead to misrepresented and unreliable conclusions when applied to the female diabetic population. Hence, female endocrinologists and researchers are encouraged to provide greater attention to conducted sex-specific studies, in order to address this issue. Recent reports state that the next generation of endocrinologists will be women, hence creating an opportunity for future researchers to address existing gender disparities, and ultimately provide quality and evidence-based medical care to all diabetic patients.

Additionally, recent trends have indicated the waning influence of insulin-related research, peaking in the 2001–2005 timeframe as depicted in the analysis, and subsequently followed by a consistent decline over the years. Moreover, our analysis failed to reveal any top 100 insulin-related article(s) published within the past decade, further identifying this declining trend. This observation may be explained by the search for a superior alternative in light of the challenges and barriers surrounding insulin therapy. Insulin, although met with significant advances over many decades, has several drawbacks such as stability issues, dosage precision, immunological aspects, reproducibility and long-term safety [42]. This led to the justified search for a newer, efficacious, and safer alternative, namely: incretin mimetics, as exemplified by the annual trends of our bibliometric analysis. As discussed previously, incretins have been observed to show a role in treating pre-diabetes, while also associated with additional advantages such as weight loss and cardiovascular benefits [24,25]. Generally, it is desirable to witness the development of a greater research interest within exploring incretin mimetics, where short- and long-term efficacy and safety may be scrutinized. Henceforth, the current barriers in anti-diabetic treatment may be overcome through comprehensive research efforts into newer anti-diabetic agents, such as incretins mimetics, while concurrently permitting the discovery of any potential defects within this popular regimen.

Ultimately, it remains essential to continue, encourage and promote efforts for groundbreaking research within the dynamic field of anti-diabetic therapies, especially with respect to newer agents, due to the observable tendency of receiving higher interest and citation count in comparison to traditional modalities, e.g., insulin or sulfonylureas.

## Limitations

Bias exists in all studies, and although we endeavored to reduce the risk of bias in our bibliometric analysis, there are certain limitations that must be highlighted. Our first limitation involves the use of Scopus as our primary search database. Research has suggested that Scopus has shown tendencies toward missing older citations, which may cause the exclusion of certain articles published before 1980 from the list [43]. Additionally, rising influential articles still require a considerable period to enter the list of the 100 most-cited articles; henceforth, future high-impact articles published currently would have yet to make it into our list. Furthermore, the presence of self-citations may lead to a considerable amount of bias in bibliometric analyses. However, our study illustrated a self-citation rate of 3.5%, which is remarkably lower than the average within the field of medicine (6.5%) [44]. The issue of self-citation can be addressed with use of the novel Eigenfactor score, a journal-level metric which employs a holistic scoring system that takes into consideration the “citedness” of a journal, while excluding the rate of self-citation within published articles. In opposition to impact factor and CiteScore, this new metric endeavors to provide an unbiased, reliable scale of the overall quality of articles published within a journal, and utilization is subsequently encouraged for future bibliometric analyses within the field of endocrinology. Finally, our analysis relied on the use of Scopus as the source of all articles, which may result in the exclusion of certain articles that have yet to be indexed or recognized by Scopus.

## Conclusion

In this bibliometric analysis, we present a comprehensive evaluation of the 100 most cited articles on anti-diabetic drugs, which were carefully selected from the available literature, providing insight into different trends in research conducted on these drugs which can serve as a benchmark for future scientific work in the field. For instance, our analysis has shown that incretin mimetics are now widely used to treat prediabetes in addition to metformin, suggesting the potential benefits of multimodality therapy. With diabetes rising in developing countries and being linked to specific ethnic groups, the need for the concerned healthcare sectors to analyze the contributing factors and set up preventive measures has also been highlighted. Moreover, most articles' authors were predominantly male, raising concerns about gender disparity. That may be due to countless reasons, including a lack of institutional opportunities and mentoring for female researchers. However, we hope to see a shift in these trends to eliminate concerns about inequity in the research sector by investigating underlying policies and ensuring that all researchers are granted equal opportunities based primarily on their expertise. Most of the articles included in the list were privately funded, introducing a potential source of bias as outcomes concerning the sponsors' interests were more likely to be published. Another source of bias found was the high number of articles with authors that had conflicts of interests also adding to the discussion on funding and sponsor's interest within the anti-diabetic research field. We hope that more fruitful discussions will take place to further improve the anti-diabetic research field.

## Supplementary Material

Supplementary Figures S1-S3 and Tables S1-S5
