# Supplementary material for: A bibliometric analysis of the 100 most-influential papers in the field of anti-diabetic drugs
Source: Future Sci OA. 2024 May 15;10(1):FSO953. doi: 10.2144/fsoa-2023-0230 (PMC11137835; doi:10.2144/fsoa-2023-0230)
Supplement: Supplementary Figures S1-S3 and Tables S1-S5 [file IFSO_A_2340880_SM0001.docx]

**Supplementary Tables & Figures**

| **Supplementary Table 1.** Top 100 original articles, their citations, and citations per year | | | |
| --- | --- | --- | --- |
| **Rank** | **Article** | **Total citations** | **Average citations per year** |
| 1 | The Diabetes Control and Complications Trial Research Group. The Effect of Intensive Treatment of Diabetes on the Development and Progression of Long-Term Complications in Insulin-Dependent Diabetes Mellitus. New England Journal of Medicine. 1993;329(14):977-86. | 22496 | 775.7 |
| 2 | Turner R. Intensive blood-glucose control with sulphonylureas or insulin compared with conventional treatment and risk of complications in patients with type 2 diabetes (UKPDS 33). Lancet. | 18800 | 783.3 |
| 3 | Knowler WC, Barrett-Connor E, Fowler SE, Hamman RF, Lachin JM, Walker EA, et al. Reduction in the incidence of type 2 diabetes with lifestyle intervention or metformin. New England Journal of Medicine. 2002;346(6):393-403. | 14324 | 716.2 |
| 4 | Van Den Berghe G, Wouters P, Weekers F, Verwaest C, Bruyninckx F, Schetz M, et al. Intensive insulin therapy in critically ill patients. New England Journal of Medicine. 2001;345(19):1359-67. | 8106 | 386 |
| 5 | Turner R. Effect of intensive blood-glucose control with metformin on complications in overweight patients with type 2 diabetes (UKPDS 34). Lancet. 1998;352(9131):854-65. | 7396 | 308.2 |
| 6 | Zinman B, Wanner C, Lachin JM, Fitchett D, Bluhmki E, Hantel S, et al. Empagliflozin, cardiovascular outcomes, and mortality in type 2 diabetes. New England Journal of Medicine. 2015;373(22):2117-28. | 6782 | 968.9 |
| 7 | Gerstein HC, Miller ME, Byington RP, Goff DC, Jr., Bigger JT, Buse JB, et al. Effects of intensive glucose lowering in type 2 diabetes. New England Journal of Medicine. 2008;358(24):2545-59. | 6376 | 455.4 |
| 8 | Patel A, MacMahon S, Chalmers J, Neal B, Billot L, Woodward M, et al. Intensive blood glucose control and vascular outcomes in patients with type 2 diabetes. New England Journal of Medicine. 2008;358(24):2560-72. | 5875 | 419.6 |
| 9 | Holman RR, Paul SK, Bethel MA, Matthews DR, Neil HAW. 10-Year follow-up of intensive glucose control in type 2 diabetes. New England Journal of Medicine. 2008;359(15):1577-89. | 5141 | 367.2 |
| 10 | Nathan DM, Cleary PA, Backlund JYC, Genuth SM, Lachin JM, Orchard TJ, et al. Intensive diabetes treatment and cardiovascular disease in patients with type 1 diabetes. New England Journal of Medicine. 2005;353(25):2643-53. | 4090 | 240.6 |
| 11 | Marso SP, Daniels GH, Frandsen KB, Kristensen P, Mann JFE, Nauck MA, et al. Liraglutide and cardiovascular outcomes in type 2 diabetes. New England Journal of Medicine. 2016;375(4):311-22. | 3975 | 662.5 |
| 12 | Steppan CM, Bailey ST, Bhat S, Brown EJ, Banerjee RR, Wright CM, et al. The hormone resistin links obesity to diabetes. Nature. 2001;409(6818):307-12. | 3911 | 186.2 |
| 13 | Duckworth W, Abraira C, Moritz T, Reda D, Emanuele N, Reaven PD, et al. Glucose control and vascular complications in veterans with type 2 diabetes. New England Journal of Medicine. 2009;360(2):129-39. | 3816 | 293.5 |
| 14 | Dormandy JA, Charbonnel B, Eckland DJ, Erdmann E, Massi-Benedetti M, Moules IK, et al. Secondary prevention of macrovascular events in patients with type 2 diabetes in the PROactive Study (PROspective pioglitAzone Clinical Trial in macroVascular Events): A randomised controlled trial. Lancet. 2005;366(9493):1279-89. | 3647 | 214.5 |
| 15 | Baur JA, Pearson KJ, Price NL, Jamieson HA, Lerin C, Kalra A, et al. Resveratrol improves health and survival of mice on a high-calorie diet. Nature. 2006;444(7117):337-42. | 3615 | 225.9 |
| 16 | Lehmann JM, Moore LB, Smith-Oliver TA, Wilkison WO, Willson TM, Kliewer SA. An antidiabetic thiazolidinedione is a high affinity ligand for peroxisome proliferator-activated receptor γ (PPARγ). Journal of Biological Chemistry. 1995;270(22):12953-6. | 3443 | 127.5 |
| 17 | Van Den Berghe G, Wilmer A, Hermans G, Meersseman W, Wouters PJ, Milants I, et al. Intensive insulin therapy in the medical ICU. New England Journal of Medicine. 2006;354(5):449-61. | 2928 | 183 |
| 18 | Ohkubo Y, Kishikawa H, Araki E, Miyata T, Isami S, Motoyoshi S, et al. Intensive insulin therapy prevents the progression of diabetic microvascular complications in Japanese patients with non-insulin-dependent diabetes mellitus: a randomized prospective 6-year study. Diabetes Research and Clinical Practice. 1995;28(2):103-17. | 2814 | 104.2 |
| 19 | Marso SP, Bain SC, Consoli A, Eliaschewitz FG, Jodar E, Leiter LA, et al. Semaglutide and cardiovascular outcomes in patients with type 2 diabetes. New England Journal of Medicine. 2016;375(19):1834-44. | 2793 | 465.5 |
| 20 | Wiviott SDSbhe, Raz I, Bonaca MP, Mosenzon O, Kato ET, Cahn A, et al. Dapagliflozin and cardiovascular outcomes in type 2 diabetes. New England Journal of Medicine. 2019;380(4):347-57. | 2738 | 912.7 |
| 21 | Scirica BM, Bhatt DL, Braunwald E, Steg PG, Davidson J, Hirshberg B, et al. Saxagliptin and cardiovascular outcomes in patients with type 2 diabetes mellitus. New England Journal of Medicine. 2013;369(14):1317-26. | 2670 | 296.7 |
| 22 | Kahn SE, Haffner SM, Heise MA, Herman WH, Holman RR, Jones NP, et al. Glycemic durability of rosiglitazone, metformin, or glyburide monotherapy. New England Journal of Medicine. 2006;355(23):2427-43. | 2543 | 158.9 |
| 23 | McMurray JJV, Solomon SD, Inzucchi SE, Kober L, Kosiborod MN, Martinez FA, et al. Dapagliflozin in patients with heart failure and reduced ejection fraction. New England Journal of Medicine. 2019;381(21):1995-2008. | 2445 | 815 |
| 24 | Crowther CA, Hiller JE, Moss JR, McPhee AJ, Jeffries WS, Robinson JS. Effect of treatment of gestational diabetes mellitus on pregnancy outcomes. New England Journal of Medicine. 2005;352(24):2477-86. | 2376 | 139.8 |
| 25 | Perkovic V, Jardine MJ, Neal B, Bompoint S, Heerspink HJL, Charytan DM, et al. Canagliflozin and renal outcomes in type 2 diabetes and nephropathy. New England Journal of Medicine. 2019;380(24):2295-306. | 2351 | 783.7 |
| 26 | Brunkhorst FM, Engel C, Bloos F, Meier-Hellmann A, Ragaller M, Weiler N, et al. Intensive insulin therapy and pentastarch resuscitation in severe sepsis. New England Journal of Medicine. 2008;358(2):125-39. | 2322 | 165.9 |
| 27 | Sanyal AJ, Chalasani N, Kowdley KV, McCullough A, Diehl AM, Bass NM, et al. Pioglitazone, vitamin E, or placebo for nonalcoholic steatohepatitis. New England Journal of Medicine. 2010;362(18):1675-85 | 2168 | 180.7 |
| 28 | Turner RC, Cull CA, Frighi V, Holman RR. Glycemic control with diet, sulfonylurea, metformin, or insulin in patients with type 2 diabetes mellitus. Progressive requirement for multiple therapies (UKPDS 49). Journal of the American Medical Association. 1999;281(21):2005-12. | 2138 | 93 |
| 29 | Chiasson JL, Josse RG, Gomis R, Hanefeld M, Karasik A, Laakso M. Acarbose for prevention of type 2 diabetes mellitus: The STOP-NIDDM randomised trial. Lancet. 2002;359(9323):2072-7. | 2124 | 106.2 |
| 30 | Neal B, Perkovic V, Mahaffey KW, De Zeeuw D, Fulcher G, Erondu N, et al. Canagliflozin and cardiovascular and renal events in type 2 diabetes. New England Journal of Medicine. 2017;377(7):644-57. | 2035 | 407 |
| 31 | Diabetes Prevention Program Research G. 10-year follow-up of diabetes incidence and weight loss in the Diabetes Prevention Program Outcomes Study. The Lancet. 2009;374(9702):1677-86. | 2009 | 154.5 |
| 32 | White WB, Cannon CP, Heller SR, Nissen SE, Bergenstal RM, Bakris GL, et al. Alogliptin after acute coronary syndrome in patients with type 2 diabetes. New England Journal of Medicine. 2013;369(14):1327-35. | 1999 | 222.1 |
| 33 | Wanner C, Inzucchi SE, Lachin JM, Fitchett D, Von Eynatten M, Mattheus M, et al. Empagliflozin and progression of kidney disease in type 2 diabetes. New England Journal of Medicine. 2016;375(4):323-34. | 1922 | 320.3 |
| 34 | Green JB, Bethel MA, Armstrong PW, Buse JB, Engel SS, Garg J, et al. Effect of sitagliptin on cardiovascular outcomes in type 2 diabetes. New England Journal of Medicine. 2015;373(3):232-42. | 1904 | 272 |
| 35 | Ott A, Stolk RP, Van Harskamp F, Pols HAP, Hofman A, Breteler MMB. Diabetes mellitus and the risk of dementia: The Rotterdam Study. Neurology. 1999;53(9):1937-42. | 1685 | 73.3 |
| 36 | Owen MR, Doran E, Halestrap AP. Evidence that metformin exerts its anti-diabetic effects through inhibition of complex 1 of the mitochondrial respiratory chain. Biochemical Journal. 2000;348(3):607-14. | 1589 | 72.2 |
| 37 | Kannel WB, Hjortland M, Castelli WP. Role of diabetes in congestive heart failure: The Framingham study. The American Journal of Cardiology. 1974;34(1):29-34. | 1583 | 33 |
| 38 | Gerstein HC, Yusuf S, Holman RR, Bosch J, Anand S, Avezum A, et al. Effect of rosiglitazone on the frequency of diabetes in patients with impaired glucose tolerance or impaired fasting glucose: A randomised controlled trial. Lancet. 2006;368(9541):1096-105. | 1543 | 96.4 |
| 39 | Shaw RJ, Lamia KA, Vasquez D, Koo SH, Bardeesy N, DePinho RA, et al. Medicine: The kinase LKB1 mediates glucose homeostasis in liver and therapeutic effects of metformin. Science. 2005;310(5754):1642-6. | 1537 | 90.4 |
| 40 | Pfeffer MA, Claggett B, Diaz R, Dickstein K, Gerstein HC, Køber LV, et al. Lixisenatide in patients with type 2 diabetes and acute coronary syndrome. New England Journal of Medicine. 2015;373(23):2247-57. | 1519 | 217 |
| 41 | Landon MB, Spong CY, Thom E, Carpenter MW, Ramin SM, Casey B, et al. A multicenter, randomized trial of treatment for mild gestational diabetes. New England Journal of Medicine. 2009;361(14):1339-48 | 1507 | 115.9 |
| 42 | Frye RL, August P, Brooks MM, Hardison RM, Kelsey SF, MacGregor JM, et al. A randomized trial of therapies for type 2 diabetes and coronary artery disease. New England Journal of Medicine. 2009;360(24):2503-15. | 1507 | 115.9 |
| 43 | Kreymann B, Ghatei MA, Williams G, Bloom SR. GLUCAGON-LIKE PEPTIDE-1 7-36: A PHYSIOLOGICAL INCRETIN IN MAN. The Lancet. 1987;330(8571):1300-4. | 1504 | 43 |
| 44 | Chiasson JL, Josse RG, Gomis R, Hanefeld M, Karasik A, Laakso M. Acarbose Treatment and the Risk of Cardiovascular Disease and Hypertension in Patients with Impaired Glucose Tolerance: The STOP-NIDDM Trial. JAMA. 2003;290(4):486-94. | 1481 | 77.9 |
| 45 | The Diabetes Control and Complications Trial Research Group. Effect of intensive diabetes treatment on the development and progression of long-term complications in adolescents with insulin-dependent diabetes mellitus: Diabetes Control and Complications Trial. The Journal of Pediatrics. 1994;125(2):177-88. | 1468 | 52.4 |
| 46 | Klein R, Klein BEK, Moss SE, Davis MD, Demets DL. The Wisconsin Epidemiologic Study of Diabetic Retinopathy: II. Prevalence and Risk of Diabetic Retinopathy When Age at Diagnosis is Less than 30 Years. Archives of Ophthalmology. 1984;102(4):520-6. | 1444 | 38 |
| 47 | Garcia MJ, McNamara PM, Gordon T, Kannell WB. Morbidity and mortality in diabetics in the Framingham population. Sixteen year follow up study. Diabetes. 1974;23(2):105-11. | 1430 | 29.8 |
| 48 | Packer M, Anker SD, Butler J, Filippatos G, Pocock SJ, Carson P, et al. Cardiovascular and renal outcomes with empagliflozin in heart failure. New England Journal of Medicine. 2020;383(15):1413-24 | 1412 | 706 |
| 49 | Larsen CM, Faulenbach M, Vaag A, Vølund A, Ehses JA, Seifert B, et al. Interleukin-1-receptor antagonist in type 2 diabetes mellitus. New England Journal of Medicine. 2007;356(15):1517-26. | 1398 | 93.2 |
| 50 | Lachin JM, Genuth S, Cleary P, Davis MD, Nathan DM. Retinopathy and nephropathy in patients with type I diabetes four years after a trial of intensive therapy. New England Journal of Medicine. 2000;342(6):381-9. | 1387 | 63 |
| 51 | Riddle MC, Rosenstock J, Gerich J. The Treat-to-Target Trial: Randomized addition of glargine or human NPH insulin to oral therapy of type 2 diabetic patients. Diabetes Care. 2003;26(11):3080-6. | 1384 | 72.8 |
| 52 | Belfort R, Harrison SA, Brown K, Darland C, Finch J, Hardies J, et al. A placebo-controlled trial of pioglitazone in subjects with nonalcoholic steatohepatitis. New England Journal of Medicine. 2006;355(22):2297-307. | 1379 | 86.2 |
| 53 | Malmberg K, Rydén L, Efendic S, Herlitz J, Nicol P, Waldenstrom A, et al. Randomized trial of insulin-glucose infusion followed by subcutaneous insulin treatment in diabetic patients with acute myocardial infarction (DIGAMI study): Effects on mortality at 1 year. Journal of the American College of Cardiology. 1995;26(1):57-65. | 1375 | 50.9 |
| 54 | DeFronzo RA, Jacot E, Jequier E, Maeder E, Wahren J, Felber JP. The effect of insulin on the disposal of intravenous glucose. Results from indirect calorimetry and hepatic and femoral venous catheterization. Diabetes. 1981;30(12):1000-7. | 1373 | 33.5 |
| 55 | Nauck MA, Heimesaat MM, Orskov C, Holst JJ, Ebert R, Creutzfeldt W. Preserved incretin activity of glucagon-like peptide 1 [7-36 amide] but not of synthetic human gastric inhibitory polypeptide in patients with type- 2 diabetes mellitus. Journal of Clinical Investigation. 1993;91(1):301-7. | 1368 | 47.2 |
| 56 | Schauer PR, Bhatt DL, Kirwan JP, Wolski K, Aminian A, Brethauer SA, et al. Bariatric surgery versus intensive medical therapy for diabetes - 5-year outcomes. New England Journal of Medicine. 2017;376(7):641-51. | 1346 | 269.2 |
| 57 | Srinivasan K, Viswanad B, Asrat L, Kaul CL, Ramarao P. Combination of high-fat diet-fed and low-dose streptozotocin-treated rat: A model for type 2 diabetes and pharmacological screening. Pharmacological Research. 2005;52(4):313-20. | 1331 | 78.3 |
| 58 | DeFronzo RA, Ratner RE, Han J, Kim DD, Fineman MS, Baron AD. Effects of exenatide (exendin-4) on glycemic control and weight over 30 weeks in metformin-treated patients with type 2. Diabetes Care. 2005;28(5):1092-100. | 1325 | 77.9 |
| 59 | Mingrone G, Panunzi S, De Gaetano A, Guidone C, Iaconelli A, Leccesi L, et al. Bariatric surgery versus conventional medical therapy for type 2 diabetes. New England Journal of Medicine. 2012;366(17):1577-85. | 1321 | 132.1 |
| 60 | Ramachandran A, Snehalatha C, Mary S, Mukesh B, Bhaskar AD, Vijay V. The Indian Diabetes Prevention Programme shows that lifestyle modification and metformin prevent type 2 diabetes in Asian Indian subjects with impaired glucose tolerance (IDPP-1). Diabetologia. 2006;49(2):289-97. | 1304 | 81.5 |
| 61 | Buse JB, Rosenstock J, Sesti G, Schmidt WE, Montanya E, Brett JH, et al. Liraglutide once a day versus exenatide twice a day for type 2 diabetes: a 26-week randomised, parallel-group, multinational, open-label trial (LEAD-6). The Lancet. 2009;374(9683):39-47. | 1254 | 96.5 |
| 62 | Gerstein HC, Bosch J, Dagenais GR, Díaz R, Jung H, Maggioni AP, et al. Basal insulin and cardiovascular and other outcomes in dysglycemia. New England Journal of Medicine. 2012;367(4):319-28. | 1236 | 123.6 |
| 63 | Malmberg K. Prospective randomised study of intensive insulin treatment on long term survival after acute myocardial infarction in patients with diabetes mellitus. British Medical Journal. 1997;314(7093):1512-5. | 1200 | 48 |
| 64 | Buchanan TA, Xiang AH, Peters RK, Kjos SL, Marroquin A, Goico J, et al. Preservation of pancreatic β-cell function and prevention of type 2 diabetes by pharmacological treatment of insulin resistance in high-risk Hispanic women. Diabetes. 2002;51(9):2796-803. | 1198 | 59.9 |
| 65 | Home PD, Pocock SJ, Beck-Nielsen H, Curtis PS, Gomis R, Hanefeld M, et al. Rosiglitazone evaluated for cardiovascular outcomes in oral agent combination therapy for type 2 diabetes (RECORD): a multicentre, randomised, open-label trial. The Lancet. 2009;373(9681):2125-35. | 1176 | 90.5 |
| 66 | Defronzo RA, Goodman AM. Efficacy of metformin in patients with non-insulin-dependent diabetes mellitus. New England Journal of Medicine. 1995;333(9):541-9. | 1162 | 43 |
| 67 | Zander M, Madsbad S, Madsen JL, Holst JJ. Effect of 6-week course of glucagon-like peptide 1 on glycaemic control, insulin sensitivity, and β-cell function in type 2 diabetes: A parallel-group study. Lancet. 2002;359(9309):824-30. | 1157 | 57.9 |
| 68 | Reichard P, Nilsson BY, Rosenqvist U. The Effect of Long-Term Intensified Insulin Treatment on the Development of Microvascular Complications of Diabetes Mellitus. New England Journal of Medicine. 1993;329(5):304-9. | 1153 | 39.8 |
| 69 | Buse JB, Henry RR, Han J, Kim DD, Fineman MS, Baron AD. Effects of exenatide (exendin-4) on glycemic control over 30 weeks in sulfonylurea-treated patients with type 2 diabetes. Diabetes Care. 2004;27(11):2628-35. | 1144 | 63.6 |
| 70 | Zoungas S, Patel A, Chalmers J, De Galan BE, Li Q, Billot L, et al. Severe hypoglycemia and risks of vascular events and death. New England Journal of Medicine. 2010;363(15):1410-8. | 1139 | 94.9 |
| 71 | Heerspink HJL, Stefánsson BV, Correa-Rotter R, Chertow GM, Greene T, Hou FF, et al. Dapagliflozin in patients with chronic kidney disease. New England Journal of Medicine. 2020;383(15):1436-46. | 1111 | 555.5 |
| 72 | Holman RR, Bethel MA, Mentz RJ, Thompson VP, Lokhnygina Y, Buse JB, et al. Effects of once-weekly exenatide on cardiovascular outcomes in type 2 diabetes. New England Journal of Medicine. 2017;377(13):1228-39. | 1104 | 220.8 |
| 73 | Flint A, Raben A, Astrup A, Holst JJ. Glucagon-like peptide 1 promotes satiety and suppresses energy intake in humans. Journal of Clinical Investigation. 1998;101(3):515-20. | 1091 | 45.5 |
| 74 | Kendall DM, Riddle MC, Rosenstock J, Zhuang D, Kim DD, Fineman MS, et al. Effects of exenatide (exendin-4) on glycemic control over 30 weeks in patients with type 2 diabetes treated with metformin and a sulfonylurea. Diabetes Care. 2005;28(5):1083-91. | 1084 | 63.8 |
| 75 | Xu G, Stoffers DA, Habener JF, Bonner-Weir S. Exendin-4 stimulates both β-cell replication and neogenesis, resulting in increased β-cell mass and improved glucose tolerance in diabetic rats. Diabetes. 1999;48(12):2270-6. | 1080 | 47 |
| 76 | Nauck M, Stöckmann F, Ebert R, Creutzfeldt W. Reduced incretin effect in Type 2 (non-insulin-dependent) diabetes. Diabetologia. 1986;29(1):46-52. | 1080 | 30 |
| 77 | Gerstein HC, Colhoun HM, Dagenais GR, Diaz R, Lakshmanan M, Pais P, et al. Dulaglutide and cardiovascular outcomes in type 2 diabetes (REWIND): a double-blind, randomised placebo-controlled trial. The Lancet. 2019;394(10193):121-30. | 1066 | 355.3 |
| 78 | The Diabetes Control and Complications Trial Research Group. Hypoglycemia in the diabetes control and complications trial. Diabetes. 1997;46(2):271-86. | 1062 | 42.5 |
| 79 | Van Den Berghe G, Wouters PJ, Bouillon R, Weekers F, Verwaest C, Schetz M, et al. Outcome benefit of intensive insulin therapy in the critically ill: Insulin dose versus glycemic control. Critical Care Medicine. 2003;31(2):359-66. | 1007 | 53 |
| 80 | Armstrong MJ, Gaunt P, Aithal GP, Barton D, Hull D, Parker R, et al. Liraglutide safety and efficacy in patients with non-alcoholic steatohepatitis (LEAN): A multicentre, double-blind, randomised, placebo-controlled phase 2 study. The Lancet. 2016;387(10019):679-90. | 993 | 165.5 |
| 81 | Pi-Sunyer X, Astrup A, Fujioka K, Greenway F, Halpern A, Krempf M, et al. A randomized, controlled trial of 3.0 mg of liraglutide in weight management. New England Journal of Medicine. 2015;373(1):11-22. | 968 | 138.3 |
| 82 | Nauck MA, Kleine N, Ørskov C, Holst JJ, Willms B, Creutzfeldt W. Normalization of fasting hyperglycaemia by exogenous glucagon-like peptide 1 (7-36 amide) in Type 2 (non-insulin-dependent) diabetic patients. Diabetologia. 1993;36(8):741-4. | 950 | 32.8 |
| 83 | Nauck M, Frid A, Hermansen K, Shah NS, Tankova T, Mitha IH, et al. Efficacy and safety comparison of liraglutide, glimepiride, and placebo, all in combination with metformin, in type 2 diabetes. Diabetes Care. 2009;32(1):84-90. | 941 | 72.4 |
| 84 | Chew EY, Ambrosius WT, Davis MD, Danis RP, Gangaputra S, Greven CM, et al. Effects of medical therapies on retinopathy progression in type 2 diabetes. New England Journal of Medicine. 2010;363(3):233-44. | 940 | 78.3 |
| 85 | Nolan JJ, Ludvik B, Beerdsen P, Joyce M, Olefsky J. Improvement in glucose tolerance and insulin resistance in obese subjects treated with troglitazone. New England Journal of Medicine. 1994;331(18):1188-93. | 917 | 32.8 |
| 86 | Garber A, Henry R, Ratner R, Garcia-Hernandez PA, Rodriguez-Pattzi H, Olvera-Alvarez I, et al. Liraglutide versus glimepiride monotherapy for type 2 diabetes (LEAD-3 Mono): a randomised, 52-week, phase III, double-blind, parallel-treatment trial. The Lancet. 2009;373(9662):473-81. | 908 | 69.8 |
| 87 | Drucker DJ, Buse JB, Taylor K, Kendall DM, Trautmann M, Zhuang D, et al. Exenatide once weekly versus twice daily for the treatment of type 2 diabetes: a randomised, open-label, non-inferiority study. The Lancet. 2008;372(9645):1240-50. | 879 | 62.8 |
| 88 | Cherney DZI, Perkins BA, Soleymanlou N, Maione M, Lai V, Lee A, et al. Renal hemodynamic effect of sodium-glucose cotransporter 2 inhibition in patients with type 1 diabetes mellitus. Circulation. 2014;129(5):587-97. | 870 | 108.8 |
| 89 | Gaede P, Vedel P, Parving HH, Pedersen O. Intensified multifactorial intervention in patients with type 2 diabetes mellitus and microalbuminuria: The Steno type 2 randomised study. Lancet. 1999;353(9153):617-22. | 859 | 37.3 |
| 90 | Haffner SM, Greenberg AS, Weston WM, Chen H, Williams K, Freed MI. Effect of rosiglitazone treatment on nontraditional markers of cardiovascular disease in patients with type 2 diabetes mellitus. Circulation. 2002;106(6):679-84. | 849 | 42.5 |
| 91 | Hernandez AF, Green JB, Janmohamed S, D'Agostino RB, Sr., Granger CB, Jones NP, et al. Albiglutide and cardiovascular outcomes in patients with type 2 diabetes and cardiovascular disease (Harmony Outcomes): a double-blind, randomised placebo-controlled trial. The Lancet. 2018;392(10157):1519-29. | 836 | 209 |
| 92 | Rohlfing CL, Wiedmeyer HM, Little RR, England JD, Tennill A, Goldstein DE. Defining the relationship between plasma glucose and HbA1c: Analysis of glucose profiles and HbA1c in the Diabetes Control and Complications Trial. Diabetes Care. 2002;25(2):275-8. | 810 | 40.5 |
| 93 | Amiel S, Beveridge S, Bradley C, Gianfrancesco C, Heller S, James P, et al. Training in flexible, intensive insulin management to enable dietary freedom in people with type 1 diabetes: Dose adjustment for normal eating (DAFNE) randomised controlled trial. British Medical Journal. 2002;325(7367):746-9. | 806 | 40.3 |
| 94 | Wu H, Esteve E, Tremaroli V, Khan MT, Caesar R, Mannerås-Holm L, et al. Metformin alters the gut microbiome of individuals with treatment-naive type 2 diabetes, contributing to the therapeutic effects of the drug. Nature Medicine. 2017;23(7):850-8. | 785 | 157 |
| 95 | Rowan JA, Hague WM, Gao W, Battin MR, Moore MP. Metformin versus insulin for the treatment of gestational diabetes. New England Journal of Medicine. 2008;358(19):2003-15. | 782 | 55.9 |
| 96 | Aschner P, Kipnes MS, Lunceford JK, Sanchez M, Mickel C, Williams-Herman DE. Effect of the dipeptidyl peptidase-4 inhibitor sitagliptin as monotherapy on glycemic control in patients with type 2 diabetes. Diabetes Care. 2006;29(12):2632-7. | 781 | 48.8 |
| 97 | Mingrone G, Panunzi S, De Gaetano A, Guidone C, Iaconelli A, Nanni G, et al. Bariatric-metabolic surgery versus conventional medical treatment in obese patients with type 2 diabetes: 5 Year follow-up of an open-label, single-centre, randomised controlled trial. The Lancet. 2015;386(9997):964-73. | 777 | 111 |
| 98 | Pearson ER, Flechtner I, Njølstad PR, Malecki MT, Flanagan SE, Larkin B, et al. Switching from insulin to oral sulfonylureas in patients with diabetes due to Kir6.2 mutations. New England Journal of Medicine. 2006;355(5):467-77. | 777 | 48.6 |
| 99 | Orchard TJ, Temprosa M, Goldberg R, Haffner S, Ratner R, Marcovina S, et al. The effect of metformin and intensive lifestyle intervention on the metabolic syndrome: The diabetes prevention program randomized trial. Annals of Internal Medicine. 2005;142(8):611-9. | 776 | 45.6 |
| 100 | Astrup A, Rössner S, Van Gaal L, Rissanen A, Niskanen L, Al Hakim M, et al. Effects of liraglutide in the treatment of obesity: a randomised, double-blind, placebo-controlled study. The Lancet. 2009;374(9701):1606-16. | 774 | 59.5 |

| Supplementary Table 2. Study types of the Articles in the top 100 list | |
| --- | --- |
| Type of Study | **Number of Articles** |
| Randomized Controlled Trial (RCT) | 84 |
| Animal experimental | 6 |
| Experimental | 4 |
| Prospective | 3 |
| Cross-sectional | 2 |
| Animal RCT | 1 |

| Supplementary Table 3. Authorship positions based on gender | | | |
| --- | --- | --- | --- |
| Variables | **Senior Author** | **First Author** | **N/A for these studies*** |
| Male | 79 | 77 | 8 |
| Female | 13 | 15 |  |
| *N/A due to the author being a group and no first or senior author was listed | | | |

| Supplementary Table 4. Top 10 most-cited review, systematic review, and meta-analysis papers in the field of anti-diabetic drugs | | | |
| --- | --- | --- | --- |
| Rank | **Article** | **Total Citations** | **Average Citations per Year** |
| 1 | Nissen SE, Wolski K. Effect of rosiglitazone on the risk of myocardial infarction and death from cardiovascular causes. N Engl J Med. 2007 Jun 14;356(24):2457-71. doi: 10.1056/NEJMoa072761. Epub 2007 May 21. Erratum in: N Engl J Med. 2007 Jul 5;357(1):100. PMID: 17517853. | 4042 | 269.5 |
| 2 | Beckman JA, Creager MA, Libby P. Diabetes and atherosclerosis: epidemiology, pathophysiology, and management. JAMA. 2002 May 15;287(19):2570-81. doi: 10.1001/jama.287.19.2570. PMID: 12020339. | 3849 | 192.5 |
| 3 | Newman DJ, Cragg GM. Natural Products as Sources of New Drugs from 1981 to 2014. J Nat Prod. 2016 Mar 25;79(3):629-61. doi: 10.1021/acs.jnatprod.5b01055. Epub 2016 Feb 7. PMID: 26852623. | 3663 | 610.5 |
| 4 | Newman DJ, Cragg GM. Natural products as sources of new drugs over the 30 years from 1981 to 2010. J Nat Prod. 2012 Mar 23;75(3):311-35. doi: 10.1021/np200906s. Epub 2012 Feb 8. PMID: 22316239; PMCID: PMC3721181. | 3524 | 352.4 |
| 5 | Drucker DJ, Nauck MA. The incretin system: glucagon-like peptide-1 receptor agonists and dipeptidyl peptidase-4 inhibitors in type 2 diabetes. Lancet. 2006 Nov 11;368(9548):1696-705. doi: 10.1016/S0140-6736(06)69705-5. PMID: 17098089. | 3007 | 187.9 |
| 6 | Pandey KB, Rizvi SI. Plant polyphenols as dietary antioxidants in human health and disease. Oxid Med Cell Longev. 2009 Nov-Dec;2(5):270-8. doi: 10.4161/oxim.2.5.9498. PMID: 20716914; PMCID: PMC2835915. | 2671 | 205.5 |
| 7 | Holst JJ. The physiology of glucagon-like peptide 1. Physiol Rev. 2007 Oct;87(4):1409-39. doi: 10.1152/physrev.00034.2006. PMID: 17928588. | 2182 | 145.5 |
| 8 | Berger J, Moller DE. The mechanisms of action of PPARs. Annu Rev Med. 2002;53:409-35. doi: 10.1146/annurev.med.53.082901.104018. PMID: 11818483. | 2060 | 103.0 |
| 9 | Yki-Järvinen H. Thiazolidinediones. N Engl J Med. 2004 Sep 9;351(11):1106-18. doi: 10.1056/NEJMra041001. PMID: 15356308. | 1875 | 104.2 |
| 10 | Bailey CJ, Turner RC. Metformin. N Engl J Med. 1996 Feb 29;334(9):574-9. doi: 10.1056/NEJM199602293340906. PMID: 8569826. | 1792 | 68.9 |

| Supplementary Table 5. Top 10 most-cited guidelines in the field of anti-diabetic drugs | | | |
| --- | --- | --- | --- |
| Rank | **Article** | **Total Citations** | **Average Citations per Year** |
| 1 | American Diabetes Association. Standards of medical care in diabetes--2014. Diabetes Care. 2014 Jan;37 Suppl 1:S14-80. doi: 10.2337/dc14-S014. PMID: 24357209. | 3623 | 452.9 |
| 2 | American Diabetes Association. Standards of medical care in diabetes--2013. Diabetes Care. 2013 Jan;36 Suppl 1(Suppl 1):S11-66. doi: 10.2337/dc13-S011. PMID: 23264422; PMCID: PMC3537269. | 3054 | 339.3 |
| 3 | American Diabetes Association. Standards of medical care in diabetes--2010. Diabetes Care. 2010 Jan;33 Suppl 1(Suppl 1):S11-61. doi: 10.2337/dc10-S011. Erratum in: Diabetes Care. 2010 Mar;33(3):692. PMID: 20042772; PMCID: PMC2797382. | 2967 | 247.3 |
| 4 | Inzucchi SE, Bergenstal RM, Buse JB, Diamant M, Ferrannini E, Nauck M, Peters AL, Tsapas A, Wender R, Matthews DR; American Diabetes Association (ADA); European Association for the Study of Diabetes (EASD). Management of hyperglycemia in type 2 diabetes: a patient-centered approach: position statement of the American Diabetes Association (ADA) and the European Association for the Study of Diabetes (EASD). Diabetes Care. 2012 Jun;35(6):1364-79. doi: 10.2337/dc12-0413. Epub 2012 Apr 19. Erratum in: Diabetes Care. 2013 Feb;36(2):490. PMID: 22517736; PMCID: PMC3357214. | 2939 | 293.9 |
| 5 | American Diabetes Association. Standards of medical care in diabetes--2011. Diabetes Care. 2011 Jan;34 Suppl 1(Suppl 1):S11-61. doi: 10.2337/dc11-S011. PMID: 21193625; PMCID: PMC3006050. | 2517 | 228.8 |
| 6 | American Diabetes Association. Standards of medical care in diabetes--2012. Diabetes Care. 2012 Jan;35 Suppl 1(Suppl 1):S11-63. doi: 10.2337/dc12-s011. PMID: 22187469; PMCID: PMC3632172. | 2276 | 227.6 |
| 7 | Inzucchi SE, Bergenstal RM, Buse JB, Diamant M, Ferrannini E, Nauck M, Peters AL, Tsapas A, Wender R, Matthews DR. Management of hyperglycemia in type 2 diabetes, 2015: a patient-centered approach: update to a position statement of the American Diabetes Association and the European Association for the Study of Diabetes. Diabetes Care. 2015 Jan;38(1):140-9. doi: 10.2337/dc14-2441. PMID: 25538310. | 2071 | 295.9 |
| 8 | Nathan DM, Buse JB, Davidson MB, Ferrannini E, Holman RR, Sherwin R, Zinman B; American Diabetes Association; European Association for Study of Diabetes. Medical management of hyperglycemia in type 2 diabetes: a consensus algorithm for the initiation and adjustment of therapy: a consensus statement of the American Diabetes Association and the European Association for the Study of Diabetes. Diabetes Care. 2009 Jan;32(1):193-203. doi: 10.2337/dc08-9025. Epub 2008 Oct 22. PMID: 18945920; PMCID: PMC2606813. | 1890 | 145.4 |
| 9 | Defronzo RA. Banting Lecture. From the triumvirate to the ominous octet: a new paradigm for the treatment of type 2 diabetes mellitus. Diabetes. 2009 Apr;58(4):773-95. doi: 10.2337/db09-9028. PMID: 19336687; PMCID: PMC2661582. | 1874 | 144.2 |
| 10 | Boyd CM, Darer J, Boult C, Fried LP, Boult L, Wu AW. Clinical practice guidelines and quality of care for older patients with multiple comorbid diseases: implications for pay for performance. JAMA. 2005 Aug 10;294(6):716-24. doi: 10.1001/jama.294.6.716. PMID: 16091574. | 1836 | 108.0 |

**Supplementary Figure 1.** Number of Publications on Insulin in Each 5-year Interval

**Supplementary Figure 2.** Number of Publications on >1 Drug Class in Each 5-year Interval

**Supplementary Figure 3.** Number of Publications on Incretin Mimetics (GLP-1 Agonists/analog) in Each 5-year Interval
